# Growth changes in infants born of adolescent mothers: Results of a national cohort study in Taiwan

**Published:** 2014-11

**Authors:** Yu-Ju Chen, Chi-Rong Li, Shu-Hsin Lee, Bu-Qing Hsu, Wei-Ya Wu, Ching-Pyng Kuo, Shiow-Li Hwang, Ming-Chih Lee

**Affiliations:** 1 *Institute of Medicine, Chung Shan Medical University (CSMU), Taichung 402, Taiwan.*; 2 *School of Nursing, CSMU, Taichung 402, Taiwan.*; 3 *Department of Nursing, CSMU Hospital, Taichung 402, Taiwan.*; 4 *School of Nursing, National Cheng-Kung University, Tainan 701, Taiwan.*; 5 *Wurih District Public Health Center, Taichung 402, Taiwan.*; 6 *National Taipei University of Nursing and Health Sciences, Taipei 365, Taiwan.*; 7 *Department of Family Medicine, Taichung Hospital, Department of Health, Executive Yuan, Taichung 402, Taiwan.*

**Keywords:** *Adolescent*, *Breastfeeding*, *Infants*, *Body weight*

## Abstract

**Background:**

Adolescent pregnancy and childbirth are associated with increased risk and challenges for both mothers and birth outcomes.

**Objective:**

To investigate the associations of growth change over time with parenting factors and to compare the differences between children born to adolescent and adult mothers in Taiwan.

**Materials and Methods:**

The dataset retrieved from Taiwan birth cohort study (TBCS) was collected by interviews using structured questionnaires, birth certificate and Passport of Well-baby Care of each child. Changes in body weight, body height and head circumference from birth to 18 months, as well as other variables were assessed by statistical analysis.

**Results:**

There were 4.13% births born to adolescent mothers in 2005. Higher ratios of breastfeeding and working were found among adult mothers (p<0.001). Significantly higher percentage of adolescent mothers caregave their infants up to 18 months (p<0.001). Children born to adolescent mothers were associated with statistically significant lower body weight (p<0.001), body height (p<0.001) and head circumference (p<0.001) in spite of velocity and slop of growth patterns were similar over time. Breastfeeding did not significantly affected growth rate during the first 6 months. Generalized estimated equation models showed that gender and preterm birth were predictive factors for birth outcomes (both p<0.001) and correlated to changes over time.

**Conclusion:**

Adolescent childbearing was associated with preterm birth and lower body weight, body height and head circumference from birth to 18 months. The changes in growth and development among children born to adolescent mothers remain to be followed and evaluated with the TBCS.

## Introduction

Pregnancy and fertility rate of teens have been decreasing in the past decade in Taiwan. Of all teens aged less than 19 who became pregnant, 20% delivered a live infant and the rate was approximately 8 births per 1000 teenage girls (ages 15-19), or 3.6% of all birth in 2005 (National Statistics, DGBES, TW) (1). Although teenage pregnancy in Taiwan is lower than western countries, such as 10% and 7% of all births in USA and the UK, the related issues have become pressing social concerns, particularly the delivery of low birth weight (LBW) or preterm infants (2, 3).

Adolescent mothers are associated with less knowledge about parenting and child development, have less confidence in their parenting abilities, and express more problematic parenting beliefs and styles when compared to adult mothers (-). There is also increased risk for premature delivery and LBW births, which could pose a threat to poorer developmental outcomes among young children (-). Children of adolescent mothers are at risk for future developmental and behavioral problems, increased risks of developmental delay, decreased self-sufficiency and continued cognitive/behavioral problems (-). Some studies have shown that factors such as greater maternal education and more favorable living conditions are likely to improve growth and developmental outcomes for those children (14).

Infant growth is not only affected by health related practices, such as breastfeeding and smoking, but also predisposing factors consisted of infant sex, birth order, and degree of intra-uterine constraint (   15 -17). Slow infant growth may indicate inadequate living conditions, inappropriate nutrition or other health problems, although factors contributing to growth vary by population such as underfeeding and infection observed in developing countries (18-20). In contrast, there is increasing evidence that rapid infant growth is associated with higher risk of developing diseases in childhood, adolescence and adulthood (-25). The pattern of infant growth has drawn attention to the effects on lifelong health (26, 27). Breastfeeding is the best way of feeding an infant and provides well-known benefits to the infant and the mother (28, 29). 

Studies have shown that total duration of breastfeeding is associated with slower growth in the first year of life and can contribute to the protective effects of breastfeeding against overweight and obesity (30-34). The World Health Organization (WHO) recommends exclusive breastfeeding (EBF) up to 6 months for newborn infants (35, 36). However, breastfeeding duration is affected by many factors, such as social status, insufficient milk supply, parity, maternal work situation and infant health problems (37, 38). In the present study we focused our investigation on the associations of growth change over time with parenting factors in a birth cohort study from Taiwan and compared the differences between children born to adolescent and adult mothers. 

## Materials and methods

**Children of 2005 birth cohort**

This was a longitudinal study using the dataset from a population-base Taiwan Birth Cohort Study (TBCS) that covered a total of 24,200 pairs of mothers and newborns from the time of birth in 2005 until December of 2007 (39). This TBCS provides a wide range of longitudinal information about children’s development. Infants with major disorders or congenital anomalies were excluded from this study. The study was reviewed and approved by the Medical Ethics Committee (National Health Research Institute) and Data Protection Board in Taiwan before initiated. After written informed consent was obtained, all eligible subjects (mother-child dyads) were categorized into two groups according to maternal ages of giving birth, of which 878 were adolescent mothers (≤20 years at the time of the child’s birth) and 20,370 were adult mothers (aged 20 or older). 

**Data collection**

Data was collected by trained research assistants with the use of a structured questionnaire at home interviews. The first and secondary home interviews with the 24,200 post-partum women were conducted at 6, 12 and 18 months after their deliveries during the period from June 2005 to December 2007. A total of 21,248 women completed interviews, giving a response rate of 87.8%. Information related to birth characteristics, including birth weight, birth order, gender, gestational age, head circumference, birth number (singleton or multiple pregnancy), method of delivery, patterns of infant feeding and caregiving status were obtained. 

All of the weight and length or height measurements were retrieved from Child Health Handbook, in which records of periodic health examinations (at 1^st^, 6^th^, 12^th^, 18^th^, 24^th^, 30^th^ and 36^th^ month) documented for all children up to 6 years old on a free-of-charge basis by National Health Insurance Program in Taiwan. The effects of breastfeeding on growth in infancy were analyzed by repeated measurements of child growth up to 18 months. The weights and lengths or heights were measured by trained nurses and recorded to the nearest 100g and millimeter (mm), respectively. Feeding practice was described by a categorical variable and included as a time-dependent variable to assess its association with growth rate.

**Statistical analysis**

Statistical analysis was performed using Statistical Analysis System (SAS) version 9.2 for Windows (SAS Institute Inc. Cary, NC, USA). Categorical variables are presented as a count and percentage and compared with a Chi-squared test or Fisher's exact test. Continuous variables are summarized by a mean and standard deviation and compared with a two-tailed independent samples t-test and results reported at a 0.05 significance level. Means, medians, standard deviations, frequencies and percentages were calculated for descriptive data. 

Trend and differences in growth for body weight, body height and head circumference between babies born to adolescent and adult mothers were analyzed using repeated measure ANOVA and Z test statistics which is comparable across ages and provides a more sensitive assessment of deviations of growth. The longitudinal associations between variables and the change of growth with time from birth to 18 months old were analyzed using fitted generalized estimating equation (GEE) models. SAS PROC GENMOD was used for logistic regression analysis to evaluate the association between growth and each of the dependent variables.

## Results

Infants born to adolescent mothers accounted for 4.13% of births in 2005 as revealed from TBCS dataset (Table I). No significant difference in the male/female ratio of infants bone to adolescent mothers was observed, however, we noted that boys were 5% higher in the group of adult mothers. Higher percentage of adolescent mothers gave birth spontaneously in comparison with adult mothers (81.0% vs. 65%, p<0.001). Although there was no statistical difference in preterm birth between two groups (p=0.306), a markedly higher incidence of LBW was observed among infants born to adolescent mothers than adult mothers (9.6% vs. 6.8%, p<0.05).

The average maternal ages at the time of giving birth of adolescent and adult mothers were 19.1±1.1 and 29.3±4.5 years, respectively. Although the vast majority of adolescent and adult mothers with singleton births stopped breastfeeding when their children were 18 months of age (96.7% and 93.5%), significant difference was detected for those were still breastfeeding between the two groups (adolescent mothers vs. adult mother: 3.3% vs. 6.5%; p<0.001). Moreover, adult mothers had significantly higher educational level in comparison to adolescent mothers (p<0.001). Higher ratio of breastfeeding and more working women were found among adult mothers at 18^th^ month (p<0.001). Conversely, 64.7% of adolescent mothers have been the main caregivers for their infants up to 18 months old, which was statistically significantly higher than adult mothers. 

**Figure 1:**
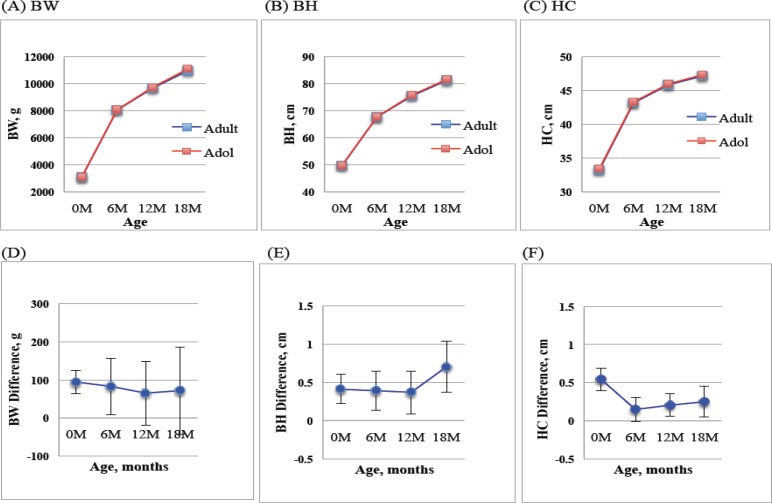
Growth trends (upper panel-A, B, C) and differences (lower panel-D, E, F) in body

The growth patterns of infants born to adolescent and adult mothers, displayed in figure 1 indicate that the velocity and slop of growth were similar in terms of body weight (BW), body height (BH) and head circumference (HC) from birth to 18 months, although the significant differences existed in each stage (Table II). The BW of infants born to adult and adolescent mothers was 3108.1±447.5 g and 3013.8±453.0 g, representing a difference of 95 g between the two groups. At the time of 18 months old, the difference decreased to 61 g. Children born to adolescent mothers were associated with statistically significant lower birth BW, BH and HC (p<0.001 for all). Trend analysis of infants between two groups showed no significant change or gain in overall weight. Although there was significant difference in changes of BH (p<0.001), no difference in overall height gain was observed. However, significant change and greater gain of HC were found in infants born to adolescent mothers (p<0.001, Table II).

**Figure 2:**
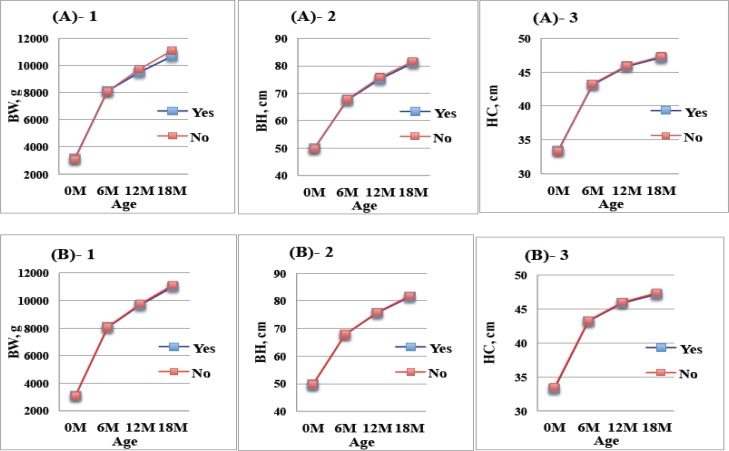
The changes in body weight (BW), body height (BH) and head circumference (HC) for children with or without breastfeeding (A) and mother caregiving at daytime (B) from birth to 18 months old.

The association between breastfeeding, daytime caregiving and growth from birth to 18 months old was assessed and depicted in Figure 2. Growth rates were significantly affected by breastfeeding in the first 6 months. Aside from both variables of breastfeeding and mother as the daytime caregiver were associated with lower growth of infants in all measures, the difference in weight velocity from 12-18 months was significant. GEE models were used to estimate the growth in terms of BW, BH and HC and the changes of growth from birth to 18 months old as functions of predictors. The results indicated that child gender, gestational age, method of delivery, and breastfeeding at 18 months were significant predictors that contributed to growth change (Table III-V). Male babies were significantly positively correlated with BW (β= 33.56, p<0.001), BH (β= 0.04, p<0.001) and HC (β= 0.04, p<0.001). The growth of preterm babies was positively correlated with age for their BW (β= 20.92, p<0.001), BH (β= 0.12, p<0.001) and HC (β= 0.07, p<0.001). 

Adolescent mothers were negatively associated with birth BW, BH and HC; however, the predictive power no longer existed over the time. Breastfeeding was negatively associated with growth change with age (p<0.001 for BW, BH and HC). Cesarean sections were positively correlated with the changes in BW (β= 2.72, p<0.05) and BH (β= 0.02, p<0.001), however, negatively correlated to HC (β= -0.03, p<0.001). The variable of mother as the primary daycare caregiver negatively contributed the change in BW (β= -4.86, p<0.001) and no significant effect on BH and HC. Multiple births and educational levels were not significantly associated with the growth changes.

**Table I:** Demographic characteristics of adolescent (n= 878) and adult (n= 20,370) mothers and their children in Taiwan

**Variables**						**Adolescent (4.13%)**	**Adult (95.87%)**	**p-value ** a
Child gender								0.226
				Male		443 (50.5%)	10702 (52.5%)	
				Female		435 (49.5%)	9668 (47.5%)	
Method of delivery								<0.001^†^
					NSD	709 (81.0%)	13231 (65.0%)	
					CS	166 (19.0%)	7128 (35.0%)	
Low birth weight (<2500 g)								<0.050 ^†^
				Yes		166 (9.6%)	7128 (6.8%)	
				No		794 (90.4%)	18995 (93.2%)	
Gestational age								0.306
			Preterm (<37 weeks)			82 (9.3%)	1703 (8.4%)	
			Full-term (≥37 weeks)			796 (90.7%)	18667 (91.6%)	
Maternal age at delivery						19.1 ± 1.1	29.3 ± 4.5	<0.111
Maternal education								<0.001^†^
			Less than high school			429 (49.1%)	2704 (13.3%)	
			High school			416 (47.6%)	8062 (39.7%)	
			College and above			29 (3.3%)	9553 (47.0%)	
Birth number								0.399
			Singleton			859 (97.8%)	19828 (97.4%)	
			Multiple			19 (2.2%)	535 (2.6%)	
Breasting at 18 months								<0.001 ^†^
			Yes			27 (3.3%)	1256 (6.5%)	
			No			784 (96.7%)	18104 (93.5%)	
Working status at 18 months								<0.001 ^†^
	Yes					369 (46.7%)	12826 (66.5%)	
	No					421 (53.3%)	6461 (33.5%)	
Caregiver during daytime at 18 months								<0.010 ^†^
		Mother				525 (64.7%)	8991 (46.4%)	
		Otherwise				286 (35.3%)	10370 (53.6%)	

NDS: normal spontaneous delivery

CS: Caesarean Section

a Bivariate analysis

† p<0.05 (Student’s *t* tests, Chi-square test)

**Table II:** Growth from birth to 18 months old of babies born to adolescent (n= 878) vs. adult (n= 20,370) mothers

**Variables**				**Adolescent (n= 878)** *	**Non-adolescent (n= 20,370)** *	**Z value ** ^a^	**p-value**
Body weight (g)							
	Birth			3013.8 ± 453.0	3108.1 ± 447.5	-6.110	<0.001^†^
	At 6 months			8002.7 ± 1049.0	8080.5 ± 1028.1	-1.977	<0.050^†^
	At 12 months			9665.4 ± 1168.2	9724.4 ± 1144.8	-1.317	0.188
	At 18 months			10992.8 ± 1323.7	11053.1 ± 1335.1	-1.004	0.316
	Change ^b^						0.129
Weight gain							
	0-6 months			4979.8 ± 987.6	4972.0 ± 947.9	0.205	0.838
	0-12 months			6634.0 ± 1095.1	6616.9 ± 1071.1	0.406	0.685
	0-18 months			7962.4 ± 1268.9	7939.8 ± 1251.1	0.400	0.689
	Change ^b^						0.877
Body Height (cm)							
	Birth			49.3 ± 2.6	49.7 ± 2.6	-4.176	<0.001 ^†^
	At 6 months			67.4 ± 3.0	67.8 ± 3.1	-3.354	<0.010 ^†^
	At 12 months			75.4 ± 3.3	75.8 ± 3.2	-3.013	<0.001 ^†^
	At 18 months			80.9 ± 3.4	81.6 ± 3.4	-4.433	<0.001 ^†^
	Change ^b^						<0.001 ^†^
Height gain							
	0-6 months			18.1 ± 3.3	18.1 ± 3.2	-0.362	0.718
	0-12 months			26.1 ± 3.5	26.1 ± 3.4	-0.284	0.776
	0-18 months			31.5 ± 3.8	31.9 ± 3.7	-2.248	<0.050^†^
	Change ^b^						0.256
Head circumference (cm)							
		Birth		32.8 ± 1.8	33.4 ± 1.7	-9.182	<0.001 ^†^
		At 6 months		43.1 ± 1.6	43.3 ± 1.7	-2.145	<0.050 ^†^
		At 12 months		45.8 ± 1.8	46.0 ± 1.7	-3.040	<0.010 ^†^
		At 18 months		47.1 ± 1.6	47.3 ± 1.8	-2.754	<0.010 ^†^
		Change ^b^					<0.001 ^†^
Circumference gain							
			0-6 months	10.2 ± 2.1	9.9 ± 2.1	4.029	<0.001 ^†^
			0-12 months	12.9 ± 2.3	12.6 ± 2.0	3.869	<0.001 ^†^
			0-18 months	14.2 ± 2.2	13.9 ± 2.2	2.118	<0.050 ^†^
			Change ^b^				<0.010 ^†^

a Z test

b Repeated measure ANOVA

† p<0.05 (ANOVA, repeated measure ANOVA)

* data are presented as mean±SD.

**Table III:** Generalized estimating equations model that assess the association between predictive factors and body weight from birth to 18 months

		**β**	**95% CI**		**X** ^2^	**p-value**
			**lower**	**upper**		
Age		886.63	883.23	890.02	262481.00	<0.001
Age^2^		-26.20	-26.35	-26.05	118868.00	<0.001
Child gender						
	Male vs. female	188.43	175.60	201.25	828.95	<0.001
Gestational age						
	Preterm vs. full-term	-608.24	-640.36	-576.12	1377.20	<0.001
Birth number						
	Multiple birth vs. Singleton	-6.17	-45.76	33.42	0.09	0.760
Method of delivery						
	CS vs. NSD	15.44	0.82	30.07	4.28	0.039
Maternal education					1.79	0.409
	High school vs. less than high school	4.67	-16.57	25.92	0.19	0.666
	College and above vs. less than high school	12.01	-8.57	32.59	1.31	0.253
Mother’s age, years						
	Adolescent (≤20) vs. adult (>20)	-70.03	-102.02	-38.04	18.41	<0.001
Breastfeeding at 18 months						
	Yes vs. no	74.24	46.15	102.33	26.84	<0.001
Daycare caregiver at months						
	Mother vs. otherwise	-4.09	-17.74	9.56	0.34	0.557
Child gender × Age		33.59	31.38	35.79	894.47	<0.001
Preterm birth × Age		20.92	16.50	25.34	86.08	<0.001
Birth number × Age		-4.95	-11.62	1.73	2.11	0.146
Method of delivery × Age		2.72	0.31	5.13	4.90	0.027
Maternal education × Age					12.26	0.002
Mother’s age × Age		3.75	-1.99	9.49	1.64	0.200
Breastfeeding at 18 months× Age		-23.34	-28.05	-18.64	94.52	<0.001
Daycare caregiver at 18 months × Age		-4.86	-7.20	-2.52	16.56	<0.001

CS: Caesarean Section

NDS: normal spontaneous delivery

CI: Confidence interval

**Table IV:** Generalized estimating equations model that assess the association between predictive factors and body height from birth to 18 months

		**β**	**95% CI**		**X** ^2^	**p-value**
			**lower**	**upper**		
Age		3.28	3.27	3.29	330603.00	<0.001
Age^2^		-0.09	-0.09	-0.09	110393.00	<0.001
Child gender						
	Male vs. female	0.86	0.79	0.93	611.59	<0.001
Gestational age						
	Preterm vs. full-term	-2.80	-2.99	-2.62	889.10	<0.001
Birth number						
	Multiple birth vs. Singleton	-0.04	-0.25	0.16	0.16	0.693
Method of delivery						
	CS vs. NSD	-0.28	-0.36	-0.21	53.55	<0.001
Maternal education					3.73	0.155
	High school vs. less than high school	0.11	0.00	0.22	3.72	0.054
	College and above vs. less than high school	0.08	-0.02	0.19	2.32	0.128
Mother’s age						
	Adolescent (≤20) vs. adult (>20)	-0.30	-0.46	-0.13	11.96	0.001
Breastfeeding at 18 months						
	Yes vs. no	0.09	-0.05	0.23	1.49	0.222
Daycare caregiver at months						
	Mother vs. otherwise	-0.02	-0.09	0.05	0.38	0.540
Child gender × Age		0.04	0.04	0.05	163.47	<0.001
Preterm birth × Age		0.12	0.11	0.13	295.75	<0.001
Birth number × Age		-0.02	-0.04	0.01	2.04	0.153
Method of delivery × Age		0.02	0.01	0.02	21.06	<0.001
Maternal education × Age					67.95	<0.001
Mother’s age × Age		0.00	-0.02	0.02	0.06	0.806
Breastfeeding at 18 months× Age		-0.04	-0.06	-0.03	41.26	<0.001
Daycare caregiver at 18 months × Age		-0.01	-0.01	0.00	2.96	0.085

CS: Caesarean Section

NDS: normal spontaneous delivery

CI: Confidence interval

**Table V:** Generalized estimating equation models that assess the association between predictive factors and head circumstance from birth to 18 months

		**β**	**95% CI**		**X** ^2^	**p-value**
			**lower**	**upper**		
Age		1.84	1.89	1.84	253674.00	<0.001
Age^2^		-0.06	-0.06	-0.06	134671.00	<0.001
Child gender						
	Male vs. female	0.60	0.55	0.64	670.16	<0.001
Gestational age						
	Preterm vs. full-term	-1.37	-1.48	-1.25	564.50	<0.001
Birth number						
	Multiple birth vs. Singleton	-0.08	-0.22	0.06	1.16	0.282
Method of delivery						
	CS vs. NSD	0.59	0.54	0.64	525.21	<0.001
Maternal education					41.36	<0.001
	High school vs. less than high school	0.22	0.14	0.29	32.55	<0.001
	College and above vs. less than high school	0.09	0.02	0.16	5.70	0.017
Mother’s age						
	Adolescent (≤20) vs. adult (˃20)	-0.24	-0.36	-0.13	17.00	<0.001
Breastfeeding at 18 months						
	Yes vs. no	0.09	0.00	0.18	4.08	0.043
Daycare caregiver at months						
	Mother vs. otherwise	-0.09	-0.14	-0.04	13.34	<0.001
Child gender × Age		0.04	0.03	0.04	332.26	<0.001
Preterm birth × Age		0.07	0.06	0.08	213.77	<0.001
Birth number × Age		0.00	-0.01	0.02	0.06	0.439
Method of delivery × Age		-0.03	-0.03	-0.02	143.19	<0.001
Maternal education × Age					0.47	0.791
Mother’s age × Age		0.01	0.00	0.03	4.60	0.032
Breastfeeding at 18 months× Age		-0.02	-0.02	-0.01	14.92	<0.001
Daycare caregiver at 18 months × Age		-0.01	0.00	0.00	0.07	0.789

CS: Caesarean Section

NDS: normal spontaneous delivery

CI: Confidence interval

## Discussion

In this study, the population-representative TBCS dataset was used to examine whether children bone to adolescent mother or adult mother were associated with growth changes over the first 18 months of life. The findings of higher incidence of LBW and prematurity among children born to adolescent mothers than their peers born to adult mothers were consistent to a previous report, although some discrepancies existed due to grouping of different age ranges (40). During the following 18 months, no significant difference in weight gain or height gain was observed; resulting in the significant differences remained. However, children of adolescent mothers not only” caught up” the growth of head circumference but even had larger size than those born to adult mothers (Table II). However, report of a systematic review indicated that early catch-up growth of infants born preterm could be associated with adverse metabolic consequences in adulthood. These infants can be shorter and lighter than term-born peers during childhood, adolescence, and adulthood, despite beneficial for neurodevelopmental outcome may be observed (41).

Our finding added to the understanding that the general higher educational level among adult mothers was likely to associate with higher breastfeeding rate, higher work engagement and lower caregiving at daytime. Furthermore, the mean age of adolescent mothers was 19±1.1 years old which was closer to adults than adolescents. They were more likely to have a wanted pregnancy and might be not associated with negative parenting behaviors in spite of lower birth outcomes.

With the use of GEE models in this study, child gender and gestational age were found to be predictive factors for birth outcomes in terms of BW, BH and HC and correlated changes over time. Adolescent mother was a predictive factor for newborns with lower BW, BH and HC. However, environmental factors, such as socioeconomic status, disadvantaged background, co-residence with other supportive family, and overall parenting quality that identified as more important predictors than maternal age for developmental outcomes in large-scale studies were not evaluated in our study (-).

According to the study of 5 birth cohorts, weight gain from 0-24 months had the strongest relationships with schooling outcomes followed by birth weight (45). The authors also suggested that growth failure in early childhood should be viewed as a marker of lack of nutrients at the cellular level that has systemic effects on growth and development in general, including the brain and neurological development. Children born at lower gestational age (32-38 weeks) and LBW were also associated with school performance, not limited to those born extremely premature (<28 weeks) or with a very low birth weight (<1500g) (46). 

Our findings indicate that the duration of breastfeeding did not contribute to greater percentage of growth, which is consistent with previous studies showing that breastfeeding is associated with slower growth in the first year of life (-). Mothers as the caregivers at daytime did not predict better infant growth in our study. Since the home environment and family resources such as mothers of adolescent mothers, adolescent fathers, family income and other determinative factors may also affect specific caregiving behaviors that were not assessed in this study, we were unable to draw further conclusion to what extent was infant growth influenced by mothers’ caregiving (47, 48).

This population-based cohort study has several strengths. The dataset provides a large national sample allowing these findings can be generalized to the entire Taiwan. The longitudinal nature of the data providing with the information over a period of time is better for assessing causal relationships than cross-sectional designs. There were some limitations of this study that must be addressed. In this paper we analyzed the physiological measurements; however, many environmental exposures (e.g. smoking, iodine deficiency and alcohol intake) and genetic variants associated with several different outcomes were not evaluated. Some potentially relevant factors, such as maternal pre-pregnancy body mass index, gestational weight gain, and gestational diabetes that may be related to postpartum growth were not available. Apart from breastfeeding, insufficient data were collected for complementary feeding, so we are unable to address potential nutritional explanations for the growth change that was manifest toward the end of the second year of life (49). Teen pregnancy and parenting remain important public health issues that deserve continued attention. 

It is more difficult for adolescent mothers to engage in a mature and sensitive manner with their children as a consequence of they face more risk factors. All of these issues can affect outcomes of the infants of adolescent mothers. The first year postpartum is a particular challenging period for adolescent mothers, as they cope with distinctive personal and social changes (50). Although children born to adolescent mothers were not at much greater risk for growth delays than their peers born to adult mothers in our study, their developmental outcomes in older ages remain unknown since we could only characterize infant growth changes in weight or length in a period of time like other studies (16, 51, 52). 
